# Pragmatic Ability Deficit in Schizophrenia and Associated Theory of Mind and Executive Function

**DOI:** 10.3389/fpsyg.2017.02164

**Published:** 2017-12-11

**Authors:** Xiaoming Li, Die Hu, Wenrui Deng, Qian Tao, Ying Hu, Xiaoxue Yang, Zheng Wang, Rui Tao, Lizhuang Yang, Xiaochu Zhang

**Affiliations:** ^1^Department of Medical Psychology, Chaohu Clinical Medical College, Anhui Medical University, Hefei, China; ^2^CAS Key Laboratory of Brain Function and Disease, School of Life Sciences, University of Science and Technology of China, Hefei, China; ^3^Department of Psychology, School of Medicine, Jinan University, Guangzhou, China; ^4^Department of Psychology, The Fourth People’s Hospital of Hefei, Hefei, China; ^5^Anhui Mental Health Center, Hefei, China; ^6^School of Humanities and Social Science, University of Science and Technology of China, Hefei, China; ^7^Center for Biomedical Engineering, University of Science and Technology of China, Hefei, China

**Keywords:** pragmatic ability, schizophrenia, theory of mind, executive function, positive symptoms, negative symptoms

## Abstract

Deficits in pragmatic abilities have frequently been observed in patients with schizophrenia. The objective of the study was to investigate the relationship between pragmatic deficits, ToM deficits and executive dysfunctions in schizophrenia. A group of 42 schizophrenic patients and 42 healthy controls were assessed on irony task (one type of pragmatic language), two subcomponents of ToM (cognitive and affective), and three subcomponents of EF (inhibition, updating, and switching). The clinical symptoms in schizophrenia were assessed using the positive and negative symptoms of schizophrenia. The schizophrenia group exhibited significant impairments in all above tasks compared to the control group. Correlation results found that irony scores were correlated with the two subcomponents of ToM and two of the three subcomponents of EF (inhibition and updating). The regression analysis revealed that the cognitive ToM and inhibition predicted 9.2% and 29.9% of the variance of irony comprehension in the patient group, and inhibition was the best predictor for performance on irony task. Irony understanding was related to positive symptoms, but not to negative symptoms. The results suggest that the ability to interpret pragmatic language depends on schizophrenic patients’ ability to infer mental states and the ability of inhibition. It provides empirical evidence for a particular target of inhibition for rehabilitation and intervention programs developed for schizophrenic patients.

## Introduction

Pragmatic skills refer to “the skills underlying competence in contextually determined, functional language use” (Turkstra et al., 1995). Thus, comprehension of pragmatic language relies on non-linguistic real-word knowledge and inference of the speaker’s beliefs and mental states in a given social context, in order to interpret utterances that convey messages that go beyond their literal meaning (Berman and Ravid, 2010). Compromised pragmatic understanding is well documented in patients with schizophrenia, and the specific manifestations include difficulties in understanding irony and metaphors (Langdon et al., 2002; Tavano et al., 2008; Sparks et al., 2010), inappropriate response to indirect request (Corcoran, 2003), difficulties in comprehension of narratives (Marini et al., 2008), and lack of coherence in discourse (Kuperberg, 2010). Utilizing the Assessment Battery for Communication (ABaCo) (Angeleri et al., 2012), Colle et al. (2013) found that patients with schizophrenia were impaired at broad pragmatic abilities compared with healthy controls, with irony understanding being the most seriously impaired. The resulting disability is major and exerts a negative impact on daily language and quality of life (Bambini et al., 2016).

Pragmatic comprehension seems to depend on the inference of the speaker’s beliefs, which is the theory of mind (ToM) ability. The label ToM, originally created by Premack and Woodruff (1978), refers to the ability to represent one’s own and other’s mental states in terms of beliefs, knowledge, or intentions, and hence to predict the agent’s behaviors. The ToM abilities are consistently found to be impaired in patients with schizophrenia (reviewed in Sprong et al., 2007; Bora et al., 2009; Bora, 2017). A strong theoretical relationship has been proposed between pragmatic comprehension and ToM in patients with schizophrenia, based on correlations between their impaired pragmatic understanding and ToM deficits (Happé, 1993; Langdon et al., 2002). For example, a previous study suggested that the mind reading capacity of patients with schizophrenia predicted their understanding of irony (Langdon et al., 2002). In addition, a number of neuroimaging studies provided evidence for overlapped neural underpinnings of ToM and irony comprehension, including the medial prefrontal cortex and temporoparietal junction (Mar, 2011; Spotorno et al., 2012; Frank et al., 2015). Most of these studies have used the ‘false belief’ paradigm and investigated cognitive ToM abilities (Shamay-Tsoory et al., 2007). However, ToM is a complex construct and can be differentiated into two subcomponents, namely, cognitive and affective (Tager-Flusberg and Sullivan, 2000; Kalbe et al., 2007; Shamay-Tsoory and Aharon-Peretz, 2007). The cognitive ToM focuses on beliefs, whereas the affective ToM focuses on emotional states and feelings (Brothers and Ring, 1992). The distinction between cognitive and affective ToM is confirmed by neuroimaging studies, which suggested that dissociated neural circuits mediate the two ToM subcomponents (Abu-Akel and Shamay-Tsoory, 2011). The cognitive ToM is mediated by a network that engages the dorsomedial and dorsolateral prefrontal cortex (Sommer et al., 2007; Kalbe et al., 2010), while the affective ToM is mediated by a network that engages the orbitofrontal cortex and ventral medial prefrontal cortex (Kipps and Hodges, 2006). The present study extended previous findings by examining the two subcomponents of ToM in schizophrenia.

Difficulties in pragmatic comprehension could also be attributed to impaired executive function (EF). The EF is a higher-order cognitive function that involves multiple processes, such as self-regulation, behavioral inhibition, shifting of cognitive sets, planning, and generation of certain behaviors (Grafman and Litvan, 1999; Miller, 2000). People rely on EF to coordinate behavior and produce flexibility in various contexts. Given that the rules of conversation continuously change with context, intact EF abilities seem necessary to enable an effective communication, particularly pragmatic understanding in certain situations (Sponheim et al., 2003; Brune and Bodenstein, 2005). Although most studies reported impaired EF and impaired pragmatic understanding in patients with schizophrenia relative to healthy controls, it is not clear whether or not executive dysfunction and pragmatic impairments are independent of one another. Previous studies investigating the relationship between EF and pragmatic ability in patients with schizophrenia reported varied results. For example, one study reported no correlation between EF and non-literal speech comprehension in schizophrenia (Langdon et al., 2002). However, another study reported a significant correlation between EF and understanding proverbs (Sponheim et al., 2003; Brune and Bodenstein, 2005). One reason for these distinct results could be that these previous studies measured the EF ability using only one single score. Indeed, confirmatory factor analysis and structural equation modeling suggested three EF subcomponents, including ‘inhibition’ (inhibition of proponent responses), ‘updating’ (information updating and monitoring), and ‘shifting’ (mental set shifting) (Miyake et al., 2000; Brydges et al., 2012). The present study aimed to explore the relationship between EF and irony understanding in schizophrenia while considering the three EF subcomponents (inhibition, updating, and shifting).

There appears to be an empirical relation between ToM and EF. A variety of theoretical accounts have been proposed (Perner and Lang, 1999; Moses and Tahiroglu, 2010), including that (a) ToM depends on EF, (b) EF is a prerequisite for the emergence of ToM, or (c) ToM tasks and EF tasks share common cognitive components. Consistent with this proposal of relation between ToM and EF, several studies reported correlations between the two in both healthy children (Sabbagh et al., 2006; Müller et al., 2012) and atypically developing children (Dennis et al., 2009; Pellicano, 2010). A review study also suggested a relationship between ToM and EF in patients with acquired neurological pathology (Aboulafia-Brakha et al., 2011). Hartwright et al. (2012) investigated the coordination of neural systems for ToM and executive control using a novel task in which psychologically relevant ToM parameters were manipulated orthogonally. The valence of these parameters modulated brain activity not only in the ToM network but also in executive control regions, suggesting that separate neural systems for executive control may support different aspects of ToM (Hartwright et al., 2012). The literature on ToM and EF in schizophrenia reported inconsistent results. Although most studies reported significant correlations between ToM and EF in patients with schizophrenia, a review study suggested independent effects of ToM and EF by reviewing eight studies using multivariate statistics (Pickup, 2008).

Our primary purpose was to explore the relationship among ToM, EF, and irony understanding. Irony is one type of pragmatic language, which conveys the exact opposite message of the literal meaning of speech (Berman and Ravid, 2010). We specifically focused on irony for three reasons. First, studies have shown that irony is frequently used in everyday discourse (Gibbs, 2000) and computer-mediated communication (Whalen et al., 2013). Second, irony is ambiguous *per se*, and it serves important communicative functions (Pexman, 2005; Gibbs and Colston, 2012), such as the expression of negative emotions and anger (Creusere, 1999; Katz et al., 2004). Third, irony comprehension has been linked to second-order ToM, whereas metaphor understanding has been linked to first-order ToM (Happé, 1995). Thus, patients with schizophrenia are more likely to be impaired at irony understanding than metaphor understanding (Happé, 1995; Langdon et al., 2002). We extended previous studies by investigating the two subcomponents of ToM (cognitive and affective ToM) and the three subcomponents of EF (updating, inhibition, and shifting) in schizophrenia. Based on the previous studies, we hypothesized that the schizophrenic group would be impaired at both cognitive and affective ToM, as well as at inhibition, updating, and shifting. We also examined whether pragmatic deficits are associated with impairments in ToM and/or EF in schizophrenia. The secondary objective of the present study was to explore further the relation between the subcomponents of ToM and EF, and the relation between irony understanding and the clinical symptoms in schizophrenia. In summary, our results indicated that patients with schizophrenia exhibited impaired pragmatic ability. The underlying impairment could be related to deficits in ToM and EF.

## Materials and Methods

### Participants

Forty-two volunteers (25 men and 17 women) diagnosed with schizophrenia based on the 10th revision of the International Statistical Classification of Diseases and Related Health Problems (ICD-10) participated in this study. They were inpatients recruited from the Center of Mental Health of Anhui province. The patients had a mean age of 26.40 (standard deviation [SD] = 5.31), with illness duration ≤ 5 years. Thirty-three patients were taking antipsychotic medications as follows: clozapine (28.57%), risperidone (16.67%), olanzapine (9.52%), chlorpromazine (9.52%), aripiprazole (7.14%), haloperidol (4.76%), and sulpiride (2.38%). A control group of 42 healthy participants were recruited through advertisement. The patient and control groups were matched for age, sex, and education level. The controls with a past or present psychiatric illness were excluded according to a semi-structured clinical interview. The exclusion criteria for patients and controls were neurological problems and visual or auditory dysfunction as assessed by chart review and consultation with the clinical staff. The study protocol was approved by the Human Ethics Committee of Anhui Medical University. Informed written consent in accordance with the Declaration of Helsinki were obtained from all participants. The patients’ clinical features and the demographic background of the two groups are summarized in **Table 1**.

**Table 1:** Clinical and demographic details of participants.

	Patients	Controls	χ^2^/*t*-values
Gender (M/F)	25/17	25/17	0.000 n.s.
Age (years) (avg ± sd)	26.40 ± 5.31	26.33 ± 5.30	0.062 n.s.
Educational level (years) (avg ± sd)	11.93 ± 1.69	11.83 ± 1.64	0.263 n.s.
Age onset illness (years) (avg ± sd)	21.84 ± 5.63		
Duration of illness (years) (avg ± sd)	2.89 ± 3.76		
PANSS Positive Score (avg ± sd)	16.40 ± 4.51		
PANSS Negative Score (avg ± sd)	19.93 ± 5.35		
PANSS General Score (avg ± sd)	34.78 ± 7.17		

*PANSS, Positive and Negative Syndrome Scale; M, male; F, female; n.s., non-significant. The χ^*2*^ test was applied to verify the independence of the gender variable of patients and healthy controls, and the *t*-test to verify the independence of the age and educational level variable.*

### Pragmatic Ability Assessment

The pragmatic ability was assessed by using irony tasks (Varga et al., 2013), with two conditions, namely, irony and control conditions. In the irony condition, the tasks consisted of a short phase describing a social situation, which was followed by an ironic statement. The control condition included tasks based on physical causality, which was not related to irony. There were 10 scenarios in each condition. The number of sentences, number of words, and grammatical complexity did not differ between the two scenarios. The experimenter read the scenarios, and the participant was asked to answer ironic or control questions after each scenario. The participant scored 1 point for a correct answer on each question; the total score for ironic questions and control questions ranged from 0 to 10.

### The ToM Assessment

The cognitive ToM was assessed by using the false-belief task based on our previous study (Li et al., 2014). The task comprised four second-order false-belief stories. Two of the stories were used by Perner and Wimmer (1985), and the other two were from the study by Sullivan et al. (1994). During the task, the experimenter read the stories and the participants were asked to answer two questions after each story. One test question was based on cognitive ToM and the other was a control question that was not related to ToM. Participants scored 1 point for a correct answer on each question; the total score for test questions and control questions ranged from 0 to 4.

The affective ToM was assessed by using an Eyes test, which comprised 30 eye photos (Baron-Cohen et al., 1997, 2001). The photos that were taken from our previous study were adapted for Chinese participants (Li et al., 2013). Each photo was accompanied by four terms, and the participant was subsequently asked to choose the term that best describes the mental state of the character in the photo. In the control condition, the participant was asked to determine the sex of each person in the photo. The participant scored 1 point for a correct response on each photo and the total score for the test questions and control questions ranged from 0 to 30.

### The EF Assessment

Inhibition was assessed by using a numerical Stroop task (Crespo et al., 2009). The task was generated using E-Prime 1.1. The task stimuli were the Arabic digits ‘2,’ ‘3,’ ‘4,’ and ‘5’ with variable length composed of two, three, four, or five digits. The stimuli were presented in black on a white background and were displayed on a 17-inch monitor of a personal computer. Two conditions were included: (1) a congruent condition in which the number of digits and identity of digits are matched, such as 22, 333, 4444, or 55555; (2) an incongruent condition in which the number of digits and identity of digits are mismatched, such as 222, 33, 44, and 555. The task consisted of two blocks, and each block contained 12 practice trials followed by 48 test trials. The congruent and incongruent trials were presented randomly in each block. The participants were instructed to report the number of digits while attempting to ignore the identity of the digit, and they were asked to respond as fast and accurately as possible. The difference in the mean reaction times between the two conditions was regarded as the index of inhibition, with lower scores indicative of superior inhibitory ability.

Updating was assessed by using a running memory paradigm (Miyake et al., 2000). The task was generated using E-Prime 1.1. The task stimuli were digits ranging from 1 to 9. Each digit was presented for 1,000 ms and a series of digits was presented at increasing length (up to 12) in the center of the screen. The participant was instructed to remember the last four digits in the sequence and to report verbally the digits when the sequence stopped. The participant was unaware of the number of digits that would be presented and of the varied length of sequences. The task consisted of 20 trials and the total score ranged between 0 and 20.

Shifting was assessed by using a digit-switching task (Salthouse et al., 1998). The task was generated using E-Prime 1.1. The task stimuli were digits ranging from 1 to 9 (excluding digit 5), which were presented in the center of the screen. The participant performed three sets of tasks in the following order: odd–even, more–less, switching. The odd–even task required the participant to press the *F* key if the digit was odd and to press the *J* key if the digit was even. The more–less task required the participants to press the *F* key if the digit was more than 5 and the *J* key if it was less than 5. The switching task required the participant to perform either the odd–even task or the more–less task according to the color of the presented digits. A red digit signaled the odd–even task, while a green digit signaled the more–less task. The participant would rely on transformation of color and task rules to complete the task. The index of shifting is the *D*-value, which is the reaction time of shifting condition minus the average reaction time for the odd–even and more–less conditions. A small score indicated strong shifting ability.

### Clinical Assessment

The participants’ clinical status was assessed using the Positive and Negative Symptoms of Schizophrenia (PANSS) (Kay et al., 1987). The PANSS is a 30-item rating instrument where each item is scored on a 7-point scale ranging from 1 (absent) to 7 (extreme). The PANSS included three sub-scales, i.e., positive symptom scale, negative symptom scale, and general psychopathology scale. Two clinical psychiatrists who were blind to the treatment status of the patients performed the assessment; their inter-rater reliability was good (Kappa > 90%).

### Statistical Analysis

The statistical analyses were carried out using SPSS 18.0 software. Group differences were assessed by using analysis of variance (ANOVA). The relationship between pragmatic ability, ToM, and EF was investigated by using the Pearson’s correlation. Hierarchical regression analyses were performed to predict the variables contributing to the pragmatic ability. The predictor variables were entered into the equations in three successive steps. In the first step, the variables of age and level of education were entered. The components of EF (inhibition, updating, and shifting) were entered in step two, and the components of ToM (cognitive ToM, affective ToM) were entered in step three.

## Results

### Pragmatic Ability Assessment

**FIGURE 1:**
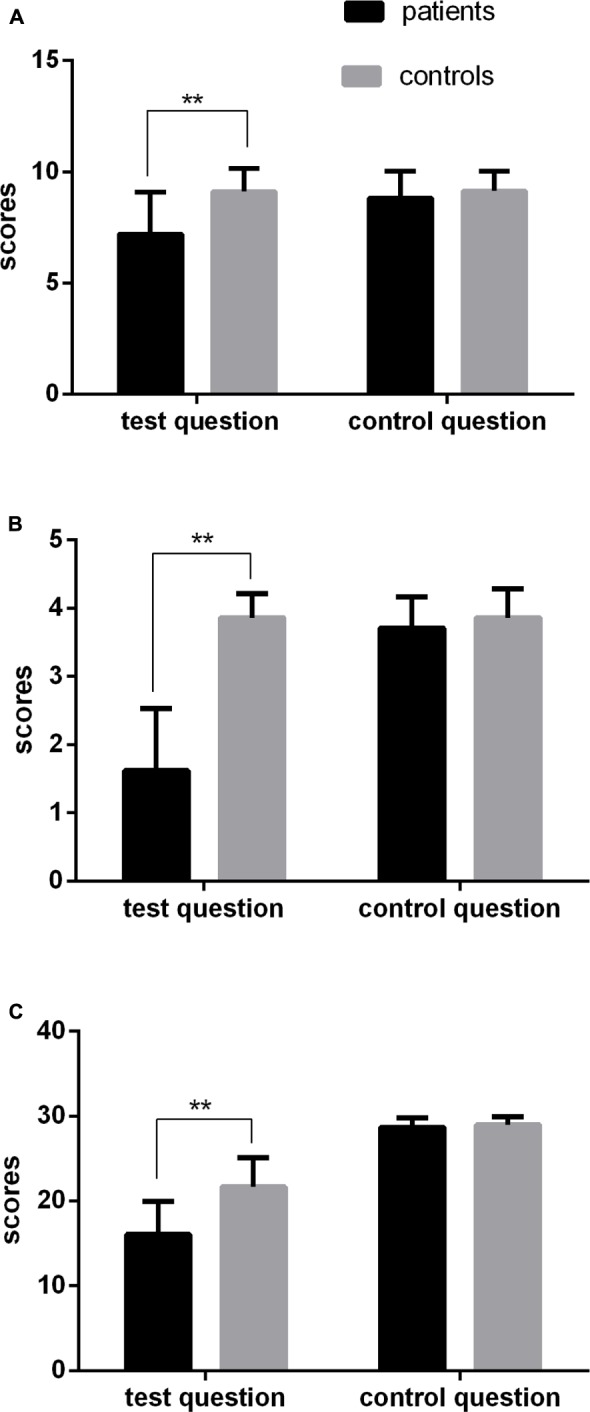
Performance on the irony tasks and two ToM tasks performed by the patient and control groups. **(A)** Group differences in the irony tasks; **(B)** Group differences in the cognitive ToM tasks; **(C)** Group differences in the affective ToM tasks. Error bars represent 1 standard error of the mean. ^∗^*pFDR* < 0.05, ^∗∗^*pFDR* < 0.01.

The scores of performance on the irony tasks were examined using a 2 (group: patient, control) × 2 (question type: test question, control question) mixed-design ANOVA. There were significant effects of group, *F*(1,82) = 17.16, *p* < 0.001, $\eta_{p}^{2}$ = 0.17, and question type, *F*(1,82) = 63.77, *p* < 0.001, $\eta_{p}^{2}$ = 0.44, with a significant interaction between them, *F*(1,82) = 60.13, *p* < 0.001, $\eta_{p}^{2}$ = 0.42. To investigate the interaction further, one-way ANOVAs were conducted on each question type. For the test question of the irony tasks, the group effect was significant, *F*(1,82) = 32.97, *p* < 0.001, $\eta_{p}^{2}$ = 0.29. The patient group had a lower score (*M* = 7.21, *SD* = 1.88) than the control group (*M* = 9.12, *SD* = 1.04). For the control question, the group effect was not significant, *F*(1,82) = 1.77, *p* > 0.05, $\eta_{p}^{2}$ = 0.02 (**Figure 1A**).

### The ToM Assessment

The scores of the cognitive ToM were examined using a 2 (group: patient, control) × 2 (question type: test question, control question) mixed-design ANOVA. There were significant effects of group, *F*(1,82) = 195.61, *p* < 0.001, $\eta_{p}^{2}$ = 0.71, and question type, *F*(1,82) = 126.80, *p* < 0.001, $\eta_{p}^{2}$ = 0.61, with a significant interaction between them, *F*(1,82) = 126.80, *p* < 0.001, $\eta_{p}^{2}$ = 0.61. To investigate the interaction further, one-way ANOVAs were conducted on each question type. For the test question of the cognitive ToM, the group effect was significant, *F*(1,82) = 220.90, *p* < 0.001, $\eta_{p}^{2}$ = 0.73. The patient group had a lower score (*M* = 1.62; *SD* = 0.91) than the control group (*M* = 3.86; *SD* = 0.35). For the control question, the group effect was not significant, *F*(1,82) = 2.24, *p* > 0.05, $\eta_{p}^{2}$ = 0.03 (**Figure 1B**).

The scores of the affective ToM were explored using a 2 (group: patient, control) × 2 (question type: test question, control question) mixed-design ANOVA. There were significant effects of group, *F*(1,82) = 44.78, *p* < 0.001, $\eta_{p}^{2}$ = 0.35, and question type, *F*(1,82) = 635.69, *p* < 0.001, $\eta_{p}^{2}$ = 0.89, with a significant interaction between them, *F*(1,82) = 44.91, *p* < 0.001, $\eta_{p}^{2}$ = 0.35. To investigate the interaction further, one-way ANOVAs were conducted on each question type. For the test question of the affective ToM, the group effect was significant, *F*(1,82) = 48.24, *p* < 0.001, $\eta_{p}^{2}$ = 0.37. The patient group had a lower score (*M* = 16.05, *SD* = 3.94) than the control group (*M* = 21.64, *SD* = 3.43). For the control question, the group effect was not significant, *F*(1,82) = 1.60, *p* > 0.05, $\eta_{p}^{2}$ = 0.02 (**Figure 1C**).

### The EF Assessment

**FIGURE 2:**
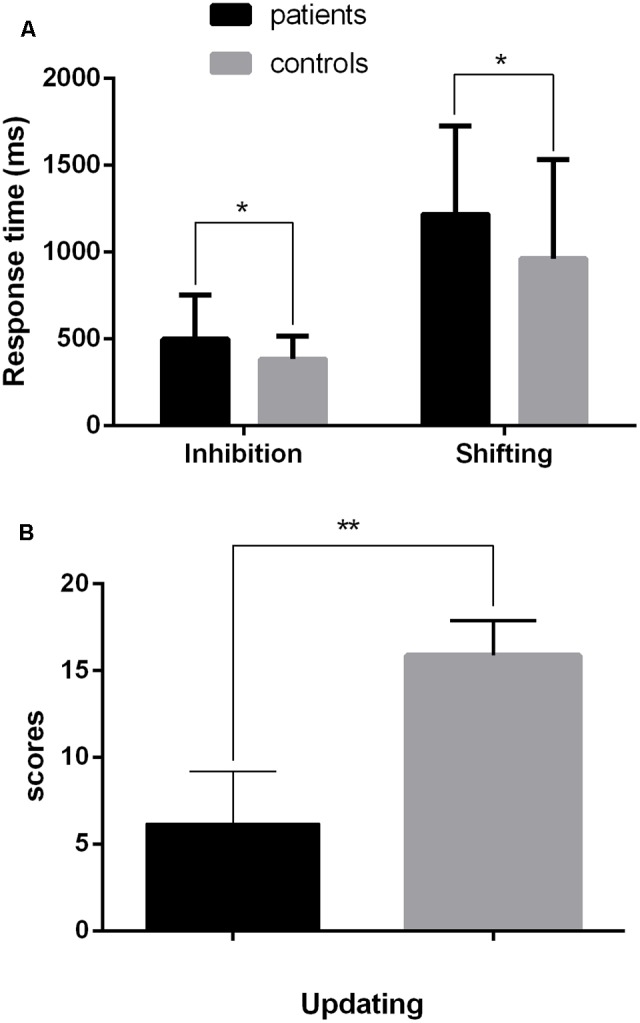
Performance on the three EF tasks performed by the patient and control groups. **(A)** Inhibition component measured by the numerical Stroop task and shifting component measured by the digit-switching task; **(B)** Updating component measured by the running memory task. Error bars represent 1 standard error of the mean. ^∗^*pFDR* < 0.05, ^∗∗^*pFDR* < 0.01.

The scores of performance on the EF tasks were measured using a 2 (group: patient, control) × 3 (task type: inhibition, updating, shifting) mixed-design ANOVA. There were significant effects of group, *F*(1,82) = 7.95, *p* < 0.006, $\eta_{p}^{2}$ = 0.09, and task type, *F*(2,164) = 222.89, *p* < 0.001, $\eta_{p}^{2}$ = 0.73, with a significant interaction between them, *F*(2,164) = 3.35, *p* < 0.05, $\eta_{p}^{2}$ = 0.04. To investigate the interaction further, one-way ANOVAs were conducted on each type of task. For the inhibition task, the effect group was significant, *F*(1,82) = 6.53, *p* = 0.012, $\eta_{p}^{2}$ = 0.07. The patient group had a poorer performance (*M* = 498.53, *SD* = 256.18) than the control group (*M* = 384.75, *SD* = 132.96). For the updating task, the group effect was significant, *F*(1,82) = 300.79, *p* < 0.001, $\eta_{p}^{2}$ = 0.79. The patient group scored less (*M* = 6.17, *SD* = 3.03) than the control group (*M* = 15.88, *SD* = 2.00). For the shifting task, the group effect was significant, *F*(1,82) = 4.64, *p* = 0.034, $\eta_{p}^{2}$ = 0.05. The patient group had a poorer performance (*M* = 1,216.69, *SD* = 509.51) than the control group (*M* = 962.00, *SD* = 572.72; **Figure 2**).

### Relationship between Pragmatic, ToM, and EF Performances

**Table 2** summarizes the Pearson correlation coefficients for pragmatic, ToM, and EF performances in the patient and control groups. For the schizophrenic group, performance on the pragmatic task was significantly correlated with the cognitive ToM, affective ToM, inhibition, and updating. For the healthy control group, performance on the pragmatic task was significantly correlated with the cognitive ToM, inhibition, updating, and shifting.

**Table 2:** Correlations between pragmatic ability scores and ToM and EF within two groups.

	Patients		Controls	
	pragmatic ability		pragmatic ability	
	*r*	*pFDR*-corrected	*r*	*pFDR*-corrected
**ToM**				
Cognitive ToM	0.451	0.007	0.429	0.005
Affective ToM	0.427	0.008	0.274	0.079
**EF**				
Inhibition	–0.709	0.000	–0.455	0.008
Updating	0.370	0.020	0.360	0.023
Shifting	–0.074	0.643	–0.516	0.000

*ToM, theory of mind; EF, executive function.*

The hierarchical regression analyses on the patient group revealed that age and educational level accounted for a largely significant proportion of the variance in pragmatic ability (adjusted *R*^2^= 0.102). In the first step, the educational level (β = 0.377, *t* = 2.229) revealed a significant influence on pragmatic ability, while age (β = 0.122, *t* = 0.898) was not significant. After controlling for the educational level, the EF subcomponents accounted for a largely significant proportion of the unique variance of pragmatic ability (Δ*R*^2^ = 0.299) in the second step. Inhibition (β = -0.627, *t* = -3,990) revealed a significant influence on the pragmatic ability, while updating (β = 0.158, *t* = 1.052) was not significant. After controlling for the educational level and inhibition, ToM explained a large significant proportion of the unique variance of pragmatic ability (Δ*R*^2^ = 0.092) in the third step. Cognitive ToM (β = 0.301, *t* = 2.331) showed a significant influence on pragmatic ability, while affective ToM (β = 0.158, *t* = 1.128) was not significant (**Table 3**).

**Table 3:** Hierarchical regression analyses predicting pragmatic ability within two groups.

	Patients		Controls	
	Pragmatic ability		Pragmatic ability	
	B	β	B	β
**Step one**				
Age	0.043	0.122	0.014	0.072
Education	0.397	0.337^∗^	0.217	0.428^∗∗^
**Step two**				
Inhibition	–0.005	–0.627^∗∗^	–0.002	–0.317^∗^
Updating	0.086	0.147	0.158	0.321^∗^
Shifting	–	–	0.000	–0.230
**Step three**				
Cognitive ToM	0.573	0.301^∗^	0.782	0.317^∗^
Affective ToM	0.075	0.158	–	–
*R*^2^ step 1		0.102		0.166
Δ*R*^2^ step 1		0.102		0.166
*R*^2^ step 2		0.401		0.478
Δ*R*^2^ step 2		0.299		0.312
*R*^2^ step 3		0.492		0.549
Δ*R*^2^ step 3		0.092		0.071

*Only variables that correlated with pragmatic ability were entered as predictors for that test. ^∗^*pFDR* < 0.05, ^∗∗^*pFDR* < 0.01.*

The hierarchical regression analyses on the control group revealed that age and educational level explained a significant proportion of the variance in pragmatic ability (adjusted *R*^2^ = 0.166). In the first step, educational level (β = 0.428, *t* = 2,864) showed a significant influence on pragmatic ability, while age (β = 0.072, *t* = 0.484) was not significant. After controlling for educational level, EF explained a large significant proportion of the unique variance of pragmatic ability (Δ*R*^2^ = 0.299) in the second step. Inhibition (β = -0.317, *t* = -2.532) and updating (β = 0.321, *t* = 2.588) showed a significant influence on pragmatic ability, while shifting (β = -0.230, *t* = -1.715) was not significant. After controlling for educational level, inhibition, and updating, ToM explained a significant proportion of the unique variance of pragmatic ability (Δ*R*^2^ = 0.092) in the third step. Cognitive ToM (β = 0.317, *t* = 2.592) showed a significant influence on pragmatic ability (**Table 3**).

### The Relationship between ToM and EF

**Table 4** summarizes the Pearson correlation coefficients for ToM and EF in the patient and control groups. Among the patients with schizophrenia, the results indicated that the inhibition was significantly correlated with both cognitive and affective ToM. No significant correlations were found between updating and the two subcomponents of ToM. The correlations between shifting and the two subcomponents of ToM also failed to reach statistical significance.

**Table 4:** Correlations for ToM and EF tasks within the groups of patients and controls.

	Patients			Controls		
	Cognitive ToM	Affective ToM	Cognitive ToM	Affective ToM	Cognitive ToM	Affective ToM
Inhibition	–0.530^∗∗^	–0.432^∗∗^	–0.693^∗∗^	–0.416^∗∗^	–0.327^∗^	–0.455^∗∗^
Updating	0.070	0.010	0.368^∗^	0.471^∗∗^	–0.132	0.360^∗^
Shifting	–0.075	0.246	–0.003	–0.424^∗∗^	–0.179	–0.516^∗∗^

*^∗^*pFDR* < 0.05, ^∗∗^*pFDR* < 0.01.*

Among the healthy participants, the results found that inhibition was significantly correlated with both cognitive and affective ToM. Updating was significantly correlated with the cognitive ToM, but not with the affective ToM. Shifting was significantly correlated with the cognitive ToM, but not with the affective ToM.

### The Relationship between Pragmatic Ability and Clinical Symptoms

The Pearson correlation coefficients revealed that the pragmatic ability was significantly correlated with the positive symptom (*r* = -0.428, *p* = 0.005), but not with the negative symptom (*r* = -0.282, *p* = 0.070).

## Discussion

The aim of the present study was to investigate the relationship between pragmatic understanding, ToM (cognitive ToM and affective ToM), and EF (inhibition, updating, shifting). As expected, patients with schizophrenia performed worse than healthy controls on the irony task. The finding is consistent with previous studies (Langdon et al., 2002; Herold et al., 2004; Langdon and Coltheart, 2004; Mo et al., 2008; Shuliang et al., 2008; Champagne-Lavau and Stip, 2010; Ziv et al., 2011). In addition, the patients were also impaired at performing the cognitive ToM, affective ToM, inhibition, updating, and shifting. Importantly, the results of the hierarchical regression analyses found that the pragmatic performance in patients with schizophrenia was predicted by the cognitive ToM and inhibition, but not by the affective ToM, updating, and shifting.

### Relationship between Pragmatic Ability and ToM Deficits

The results of the Pearson correlation analyses indicated a significant correlation between impaired understanding of irony and deficits in cognitive and affective ToM in the patient group. Similarly, cognitive and affective ToM both significantly predicted the irony understanding in patients with schizophrenia. The finding of the relation between cognitive ToM and irony understanding is consistent with previous studies that reported a relationship between cognitive ToM and pragmatic ability in schizophrenia (Langdon and Coltheart, 2004; Mazza et al., 2008; Champagne-Lavau and Stip, 2010) and other clinical situations such as right-hemisphere lesions, autism, and traumatic brain injury (Happé, 1993; Winner et al., 1998; Champagne-Lavau and Joanette, 2009). Nevertheless, one study has previously reported that pragmatic deficits were not related to cognitive ToM in schizophrenia (Mo et al., 2008). Information underlying ironic remarks is not directly stated, but merely implied. Hence, irony understanding requires first-order inference about the speaker’s belief as well as second-order inference about the speaker’s beliefs about the listener’s beliefs (Marjoram et al., 2005). Therefore, impaired understanding of irony has been associated with the cognitive ToM.

The affective ToM seems to contribute to irony understanding in schizophrenia. Verbal irony often conveys the speaker’s emotions and attitudes that are of opposite valence to the literal meaning of the words spoken. Indeed, different types of irony evoke different emotions. For instance, ironic criticism or sarcasm would elicit negative emotions such as anger, disgust, and contempt. One example is that you said to a friend “you are really good at that” when the friend failed an examination, which involves a positively worded statement that is meant to be taken negatively. The speaker’s attitudes and emotions could be inferred from their paralinguistic features, such as tone of voice, facial expression, and gestures. The ability to infer other people’s emotions and feelings is designated affective ToM (Shamay-Tsoory et al., 2009, 2010). Despite the theoretical relationship between comprehension of irony and affective ToM, only one study reported a mediation effect of affective ToM on the relation between age and understanding ironic criticism in school-aged children (Agostino et al., 2017). Although we found an association between affective ToM and irony understanding in patients with schizophrenia, the regression results suggested that affective ToM no longer predict irony performance when controlling the effect of EF. This finding shed new light on the mixed findings on the association between affective ToM and irony.

### Relationship between Pragmatic Ability and EF Deficits

We investigated the effect of EF on pragmatic ability in patients affected by schizophrenia. We found that patients were impaired in the three EF components (inhibition, shifting, and updating). Correlation analysis revealed that the ability to understand irony was correlated with the scores of inhibition, updating, but not with shifting in the patient group.

According to the psycholinguistic model of non-literal language understanding (Champagne and Joanette, 2004), irony comprehension seems to depend on the inhibition of the automatic processing and interpretation of literal meaning of an utterance. As a consequence, impaired inhibition may result in deficits in suppressing the more readily accessed literal meanings (McDonald and Pearce, 1996). Previous researchers have also reported associations between inhibition and pragmatic functions after traumatic brain injury (Channon and Watts, 2003). However, Langdon et al. (2002) found that difficulties in understanding metaphors and irony on a story-comprehension task in patients with schizophrenia were independent of inhibition.

In addition to inhibition, impaired updating was also associated with the pragmatic performance in schizophrenia. Ironic statements typically provide a direct literal meaning and an indirect non-literal message. Updating enables the coding of stimuli and replacing non-relevant information with relevant information in working memory (Morris and Jones, 1990). The present results suggested that updating plays a role in understanding irony. However, updating has been rarely examined previously, even though it is one of the most frequently studied EFs (Miyake and Friedman, 2012). To our knowledge, this is the first study that has attempted to examine the relation between irony and updating.

The present results did not find a relation between shifting and irony understanding in schizophrenia. Shifting is regarded as a form of flexibility in response to changing contexts. Previous studies suggested that flexibility did not play a role in pragmatic performance in patients with schizophrenia (Langdon et al., 2002; Champagne-Lavau and Stip, 2010), right-hemisphere lesion (Champagne-Lavau and Joanette, 2009), and traumatic brain injury (Martin and McDonald, 2005). In contrast, some studies reported a relation between reduced flexibility and impaired pragmatic performance (Sponheim et al., 2003; Brune and Bodenstein, 2005). These inconsistencies could be attributed to the varied tasks and different types of pragmatic language.

### Pragmatic Ability Predicted by ToM and Inhibition

The regression analysis indicated that the cognitive ToM explained 9.2% of the unique variance of pragmatic ability in patients with schizophrenia, while only one of the three EF components (inhibition) explained 29.9% of the unique variance. These results suggested that cognitive ToM could predict impaired irony comprehension, and that inhibition was the best predictor of the patients’ ability to comprehend irony. Channon and Watts (2003) found that three predictors of inhibitory control gave rise to a significant regression equation accounting for 36% of the variance of pragmatic comprehension in patients with traumatic brain injury. These results suggested a critical role of the inhibition abilities in irony comprehension in patients with schizophrenia. It also suggested a central role of inhibition among the three EF components (Westerhausen et al., 2011). According to the three-factor model of EF (Miyake et al., 2000), inhibition is regarded as a central sub-component of EF, which is the ability to inhibit deliberately dominant, automatic, or prepotent responses in the presence of competing sources. These inhibition features enable patients with schizophrenia to understand pragmatic language, suggesting a particular target of inhibition for rehabilitation and intervention programs.

### Relationship between ToM and EF Deficits

The regression results indicated significant correlations between inhibition and the two ToM subcomponents in both schizophrenia and healthy groups. This finding suggested that reasoning about the beliefs and emotions of others relies, at least partly, on the inhibitory processing. Two types of evidence from developmental studies on preschool children support this inhibitory account. First, the difficulty level of ToM tasks could be manipulated by modifying the inhibitory demands (Leslie et al., 2005; Yazdi et al., 2006). Second, previous studies demonstrated that children’s performance on ToM tasks is correlated with their performance on tasks assessing inhibition (Carlson and Moses, 2001; Rakoczy, 2010). In addition to inhibition, updating and shifting were correlated with cognitive ToM in healthy controls, but not in patients with schizophrenia. The finding of correlation between the two EF subcomponents (updating and shifting) and cognitive ToM in healthy individuals is consistent with previous studies (Austin et al., 2014; Li et al., 2014). Importantly, the regression results in both patients and healthy controls indicated that ToM ability continued to predict the performance on irony tasks once EF was controlled for. Thus, ToM and EF may independently contribute to the ability of understanding irony.

### Relationship between Pragmatic Ability and Symptoms

The present findings suggested that irony understanding was related to positive symptoms, but not to negative symptoms. Misunderstanding of irony may contribute to developing positive symptoms such as delusions. According to the theoretical model proposed by Salvatore et al. (2012), an alteration in pragmatic understanding of the mind of others is one of several triggers for delusional experience in schizophrenia. Furthermore, several positive symptoms such as disorganized thought and difficulty in following a conversation could also contribute to misunderstanding irony. A previous study reported a correlation between positive symptoms and pragmatic performance (Stratta et al., 2007). In contrast, another study reported a correlation between negative symptoms and pragmatic performance in schizophrenia (Saban-Bezalel and Mashal, 2017). Those inconsistencies call for future studies to clarify the relation between irony understanding and specific clinical symptoms in schizophrenia.

### Limitations

Several limitations of the present study should be considered. First, as most patients were medicated in the present study, our results may be influenced by antipsychotic effects. Second, our sample size was moderate, leading to limited statistical power for the mediation effect. Thus, the findings in this study should be considered preliminary. Moreover, there is a lack of comparison between medicated and unmediated patients due to our sample size. Third, we recruited only patients with early-stage schizophrenia. Patients with chronic schizophrenia should be evaluated in future studies. Finally, an intelligence quotient (IQ) test or a test of general cognitive ability should have been used to rule out the possibility that the worse performance of the patients compared to the controls might be explained just in terms of their different general cognitive competence; however, such tests were not included in the present study.

## Conclusion

In the present study, we aimed to explore the relationship between pragmatic ability deficit, which was assessed by using irony tasks, with ToM and EF performance in patients with schizophrenia. To the best of our knowledge, this study is the first to examine the two subcomponents of ToM (cognitive ToM and affective ToM) and the three subcomponents of EF (inhibition, shifting, and updating) in patients with schizophrenia. The results suggested that the two subcomponents of ToM and two of the three subcomponents of EF (inhibition and updating) were strongly correlated with irony understanding. In particular, the cognitive ToM and one of the three subcomponents of EF (inhibition) predicted the performance of irony comprehension in the patient group, and inhibition was the best predictor of their ability to comprehend irony. Inhibition seems to play a critical role in irony comprehension in patients with schizophrenia, potentially offering targets of therapeutic interventions in schizophrenia.

## Author Contributions

XL and XZ designed experiments. WD, YH, XY, and RT performed experiments. XL and ZW analyzed data. XL, DH, QT, LY, and XZ wrote the paper.

## Conflict of Interest Statement

The authors declare that the research was conducted in the absence of any commercial or financial relationships that could be construed as a potential conflict of interest.
